# Does one workshop on respecting cultural differences increase health professionals’ confidence to improve the care of Australian Aboriginal patients with cancer? An evaluation

**DOI:** 10.1186/s12913-017-2599-z

**Published:** 2017-09-15

**Authors:** Angela Durey, Georgia Halkett, Melissa Berg, Leanne Lester, Marion Kickett

**Affiliations:** 1 UWA Dental School, University of Western Australia, 35 Stirling Highway, Perth, Western Australia 6009 Australia; 2 Centre for Aboriginal Studies, Curtin University, Kent Street, Bentley, Perth, 6102 Western Australia Australia; 3 School of Nursing, Midwifery and Paramedicine, Curtin University, Kent Street, Bentley, Perth, 6102 Western Australia Australia; 4 Health Promotion and Evaluation Unit, University of Western Australia, 35 Stirling Highway, Perth, 6009 Western Australia Australia

**Keywords:** Aboriginal people, Education, Cancer, Cultural differences, Mixed-methods, Health professional

## Abstract

**Background:**

Aboriginal Australians have worse cancer survival rates than other Australians. Reasons include fear of a cancer diagnosis, reluctance to attend mainstream health services and discrimination from health professionals. Offering health professionals education in care focusing on Aboriginal patients’ needs is important. The aim of this paper was to evaluate whether participating in a workshop improved the confidence of radiation oncology health professionals in their knowledge, communication and ability to offer culturally safe healthcare to Aboriginal Australians with cancer.

**Methods:**

Mixed methods using pre and post workshop online surveys, and one delivered 2 months later, were evaluated. Statistical analysis determined the relative proportion of participants who changed from not at all/a little confident at baseline to fairly/extremely confident immediately and 2 months after the workshop. Factor analysis identified underlying dimensions in the items and nonparametric tests recorded changes in mean dimension scores over and between times. Qualitative data was analysed for emerging themes.

**Results:**

Fifty-nine participants attended the workshops, 39 (66% response rate) completed pre-workshop surveys, 32 (82% of study participants) completed post-workshop surveys and 25 (64% of study participants) completed surveys 2 months later. A significant increase in the proportion of attendees who reported fair/extreme confidence within 2 days of the workshop was found in nine of 14 items, which was sustained for all but one item 2 months later. Two additional items had a significant increase in the proportion of fair/extremely confident attendees 2 months post workshop compared to baseline. An exploratory factor analysis identified three dimensions: communication; relationships; and awareness. All dimensions’ mean scores significantly improved within 2 days (*p* < 0.005) and persisted to 2 months. The workshop raised awareness about barriers and enablers to delivering services respectful of cultural differences, led to a willingness to reflect on pre-existing beliefs and assumptions about Aboriginal Australians that in some cases resulted in improved care.

**Conclusion:**

Single workshops co-delivered by an Aboriginal and non-Aboriginal presenter can be effective in building health professionals’ confidence and translating into practice knowledge of respectful care of Aboriginal patients with cancer. Sustaining improvements may require integrating this approach into ongoing professional development.

**Electronic supplementary material:**

The online version of this article (10.1186/s12913-017-2599-z) contains supplementary material, which is available to authorized users.

## Background

Rates of cancer in Indigenous peoples in the developed world have been increasing compared to non-Indigenous populations, with higher prevalence of risk factors, late diagnosis, poorer adherence to treatment and lower survival rates [1]. While Australia’s rates of survival from cancer rank among the best in the world, Aboriginal and Torres Strait Islander people fare worse than other Australians. Cancer is the leading cause of death in this population group after cardiovascular disease with a 16% increase in death rates from cancer between 1998 and 2012 [2]. From 2008 to 2012, 20% of deaths in Aboriginal Australians were caused by cancer [3]. Depending on the type of cancer, there are differences in the incidence, mortality and survival of Aboriginal people compared to other Australians. Aboriginal women had a 68% higher risk of dying from breast cancer than other women in Australia after adjusting for diagnostic period and socio-demographic factors [4]. In New South Wales (NSW) Australia, incidence and mortality rates for lung cancer and cervical cancer were higher in Aboriginal people compared to the non-Aboriginal population and lower for bowel cancer and breast cancer [5]. Survival rates after 5 years for head and neck cancers in Aboriginal men in NSW were lower than for other men [5]. However, while squamous cell carcinoma was the most common malignancy affecting the oral cavity, there was no statistically significant difference in survival rates between Aboriginal and non-Aboriginal Australians living in Western Australia [6].

In this paper, we use the term “Aboriginal” to describe the local Indigenous population which is the preferred terminology used by the Western Australian (WA) Department of Health [7]. With increasing rates of co-morbidity and limited access to comprehensive culturally responsive care for Aboriginal Australians, important questions have been raised about factors influencing timely access to health services and uptake of treatment [8]. Research indicates Aboriginal beliefs and perceptions around cancer include fear it is a death sentence reflected in a sense of fatalism following diagnosis; that it is contagious and that some prefer traditional methods over a western biomedical approach [9]. A reluctance to attend services for diagnosis and treatment is compounded for those with a limited understanding of the western medical system, those living in remote areas and other factors such as racism from service providers and lack of awareness of and sensitivity to Aboriginal people’s needs and lived experience [9]. Shahid et al. [9] suggested health providers need to respond better to Aboriginal people’s needs, understand their concerns, communicate more respectfully and adopt a more holistic approach if the number of Aboriginal patients engaging with cancer treatment is to increase. This includes training in culturally safe care that is respectful of Aboriginal culture. The concept of cultural safety was developed by Irihapeti Ramsden [10] a Maori nurse in New Zealand. She recognised the role of power relations in health care where the detrimental effects of colonisation were reflected in differentials in power relations between non-Maori health professionals and Maori patients that negatively impacted on Maori health outcomes. Culturally safe care can be demonstrated in health services respectful to Aboriginal patients reflected in environmental design, employment of Aboriginal health professionals and a recognition of cultural needs so Aboriginal people feel comfortable and welcomed in a mainstream health setting [11].

A strategic obejective of the Radiation Oncology Tripartite committee, a peak group in radiation oncology in Australia, was to provide Aboriginal patients with “access to radiotherapy services offered in a culturally appropriate and respectful way” ([12], page 16) and improve recruitment and retention of radiation oncology health professionals by a range of strategies including continuing professional development programs. In 2012, a Western Australian collaborative of radiation therapists, radiation oncology medical physicists and tertiary education stakeholders, whose overall aim was sustaining the radiation oncology workforce through collaborative research and education, was successful in obtaining funding through the Better Access to Radiation Oncology Scheme. The Radiation Oncology Workforce WA group identified a number of continuing professional development opportunities for health professionals including one addressing communication, support and treatment adherence of Aboriginal Australians with cancer. This paper presents findings from an evaluation of a professional development workshop entitled, “Working together to improve cancer care for Aboriginal and Torres Strait Islander Australians”, provided once each at two different cancer treatment centres. The workshops aimed to improve the confidence of radiation oncology health professionals in their knowledge, communication and ability to offer culturally safe healthcare to Aboriginal Australians with cancer. The workshops were presented to radiation therapists, radiation oncology nurses and other radiation oncology staff by an Aboriginal (MK) and non-Aboriginal (AD) researcher.

## Methods

### Design

Pre and post online surveys were designed to evaluate the workshops with an additional 2 months follow-up survey. Both qualitative and quantitative data were collected.

### Setting and sample

The workshops were located in two sites dedicated to cancer care in Perth, Western Australia. Quality Assurance approval with the intent to publish was gained from both health providers.

Radiation oncology health professionals who registered online to attend one of the workshops were contacted by email and invited to participate in the study.

After completing an online consent form and the baseline survey, participants attended the workshop. Two days following each workshop, eligible participants who had completed a baseline survey and had attended the workshop were emailed a link to the online post-workshop survey with two further weekly email reminders to complete the survey. Two months after the workshop, participants were emailed a link to the follow up survey and received up to two further weekly email reminders to complete.

### Workshops: ‘Working together to improve cancer care for aboriginal and Torres Strait islander Australians’

The overarching principle underpinning the workshops was how Aboriginal and non-Aboriginal Australians can work together to improve healthcare for Aboriginal Australians. Both 2 hour workshops used theory, case studies and group discussions to explore barriers and facilitating factors to delivering culturally safe care to Aboriginal people with cancer. Participants were introduced to social and cultural determinants of health and power differentials underpinning theories of white racial privilege following colonisation [13, 14].

Case studies illustrating culturally unsafe care informed group discussions which actively involved participants. For example, one case study described an older Aboriginal male elder from a remote community in Western Australia who was receiving treatment from a young female non-Aboriginal nurse. Discussion of barriers and facilitating factors raised by the case study included: cultural differences in expectations of appropriate behaviour related to gender and age; medical language and using unfamiliar terminology in explanations of treatment; lack of inclusion of family members in decisions about care; and the effect of the ongoing legacy of colonisation on Aboriginal health and wellbeing reflected in research evidence of disparities in cancer care for Aboriginal compared to non-Aboriginal patients [15].

A focus of the workshop was the importance of identifying discriminatory behaviour. Attendees were provided with evidence that racism in health services persists, is often unreported and unexamined [16], and can have a negative impact on health and wellbeing [17]. This was illustrated by a case study where the western biomedical model of care with its ‘egalitarian’, one-size-fits-all approach to cancer care, was examined for how it ignored cultural differences in ways that compromised rather than promoted the health and wellbeing of Aboriginal patients [13, 14, 18, 19]. One presenter (MK), drew on her work as a registered nurse who had practised across Australia and her experience as an Aboriginal person, and that of her family, to illustrate examples of good and poor quality care delivered to Aboriginal Australians. She observed that health professionals offering health care in hospital respectful of cultural differences alleviated the patient’s fear and helped build their trust. The ensuing small group discussions amongst workshop participants explored the issues further and highlighted the importance of health providers reflecting on and questioning their own assumptions about Aboriginal people that can impact on the care they provide.

### Instruments

Three online surveys (Qualtrics, Provo, UT) were administered pre-workshop, post-workshop and 2 months follow up. All contained 14 items related to culturally safe practice with a four point rating scale where participants rated their self-perceived confidence from not at all confident (0), a little bit confident (1), fairly confident (2), and extremely confident (3). The items were adapted from an evaluation of an intercultural leadership program supporting tertiary educators teaching Indigenous health and culture to prepare interdisciplinary students to work respectfully and appropriately as health professionals with Indigenous peoples [20]. In addition, the pre workshop survey had eight demographics items and three open-ended questions about current cultural perceptions (Additional file 1).

The post-workshop survey included the 14 items related to culturally safe practice and an additional six questions to rate the workshop in terms of whether the participant’s learning needs were met and whether the activity was relevant to their own practice. Three open-ended questions were included to determine the most significant learning gained, any suggestions for improvement and further comments (Additional file 2). As this was a self-reported questionnaire, responses to open-ended questions allowed researchers to better understand whether participants’ confidence to engage with Aboriginal patients was influenced by participating in the workshop.

The 2 months follow-up survey included the 14 items related to culturally safe practice and five open-ended questions where participants were asked to: describe significant learning from the workshop and its influence on caring for Aboriginal patients; factors that have linked learning to practice; challenges in linking learning to practice and any further comments (Additional file 3).

## Data analysis

### Quantitative

All data were downloaded from Qualtrics™ to IBM SPSS Statistics (version 22) which was used for the analysis. Chi-square tests were used to test whether demographic variables were significantly different in relation to ratings of confidence (bivariate) at baseline, or in tests which had high proportions (>20%) of small expected counts Fisher’s Exact Tests (FET) were used; however, none were significant. McNemar’s related sample tests were used on each cultural competency item to compare the number of participants who changed their baseline rating from not at all/ a little bit confident to fairly/extremely confident, and vice-versa, immediately after the workshop and 2 months after the workshop. An exploratory factor analysis was used to determine underlying dimensions of the 14 cultural competency items. A mean score for each factor was calculated and graphical inspection of cultural competency confidence scores revealed skewed distributions and Kolmogorov-Smirnov tests confirmed that post workshop scores were not normally distributed. Therefore, changes over time for each factor were tested by a nonparametric Friedman’s ANOVA test. Wilcoxon tests were used for pairwise comparisons to determine differences in the mean score for each factor between time points.

### Qualitative

The qualitative data from written responses to open-ended survey questions were analysed using thematic analysis [21]. Three researchers analysed the findings; two read the qualitative responses to the surveys and independently identified the emerging themes in response to each question and the third collated these responses and organised them into key themes so similarities, differences and anomalies could be noted. Findings were then further discussed between the three researchers until consensus was reached about the emerging themes. This process ensured triangulation and provided a rigorous approach to the analysis.

## Results

Quantitative findings from the workshops are presented first followed by qualitative findings.

Of the 59 participants who registered for a workshop, 44 consented to participate in the study and completed the online pre-workshop survey. Thirty-nine participants (66% of total workshop registrants (*n* = 59)) were included in the study as they consented to participate, completed a baseline survey, and actually attended one workshop. Twenty-two participants attended the workshop at Site A (56%) and 17 at Site B (44%). Of 39 study participants, 32 completed a post-workshop survey (82%) and 25 completed a 2 months post workshop survey (64%). At baseline, one participant completed only 6 scale items and one participant completed only open-ended questions, and their missing data prevented inclusion in statistical time point comparisons. One participant completed only open-ended questions in the post-workshop survey.

Demographic characteristics are noted in Table 1. Most participants were radiation therapists (RTs) or postgraduate RT students, approximately half were aged 30 years or older (54%) and had at least 10 years’ experience (52%). Consistent with the proportion of female medical radiation practitioners in Australia in the period 2013–2014 (67%) ([22], page 246), the majority of participants in this study were female (82%). Significantly more participants at Site B were older than 30 years (71%) compared to participants at Site A (27%)(*X* ^2^ [1, *N* = 39] = 7.240, *p* = 0.007). There were no other significant differences between participants at each site for demographic characteristics or item ratings.

**Table 1:** Personal profile characteristics and qualifications of education workshop attendees (*n* = 39)

	*n* (%)
Age	
< =30 years	21 (53.8)
> 30 years	18 (46.2)
Gender^a^	
Male	6 (15.4)
Female	32 (82.1)
Profession	
Radiation Therapist (RT)	25 (64.1)
Radiation Oncology Nurse (RON)	1 (2.6)
Student Master of RT (Student RT)	9 (23.1)
Other	4 (10.3)
Qualification^a^	
Certificate	1 (2.6)
Diploma	7 (17.9)
Bachelor degree	23 (59.0)
Master degree	5 (12.8)
Other	2 (5.1)
Number of years practicing^a^	
≤ 10 years	15 (51.7)
> 10 years	14 (48.3)
Location trained	
WA	19 (48.7)
Other Australian State	6 (15.4)
Overseas	14 (35.9)
Aboriginal and/or Torres Strait Islander patients in case load	
Yes	28 (71.8)
No	6 (15.4)
Don’t know	5 (12.8)
Undergone prior Aboriginal cultural education and training	
Yes	3 (7.7)
No	36 (92.3)

^a^contains missing data

### Sensitivity analysis

A sensitivity analysis was performed on responses of participants who dropped out of later surveys. Participants who did not complete a post-workshop survey were significantly less likely to be fairly/extremely confident at baseline about “interacting with people from Aboriginal or Torres Strait Islander cultures” (29%), compared to participants who completed a post-workshop survey (81%)(FET, *p* = 0.014), and were significantly less likely to be fairly/extremely confident at baseline about initiating “conversations with people from Aboriginal or Torres Strait Islander cultures” (29%) compared to participants who completed a post workshop survey (74%)(FET, *p* = 0.034). Similarly, participants who did not complete a post workshop survey scored significantly lower for the Communication sub-scale at baseline (Mdn = 1.0) compared with those who completed the post workshop survey (Mdn = 2.0)(*U* = 164.00, *z* = 2.163, *p* = 0.036). Baseline sub-scale scores of participants who completed a post workshop survey and those who did not were not significantly different for the Relationships (*U* = 121.00, z = 1.160, *p* = 0.265) or Awareness sub-scales (*U* = 96.50, *z* = −0.458, *p* = 0.658). There were no significant differences in responses to any cultural competency items at baseline in participants who completed the 2 months follow-up survey and those who did not. Scores were not significantly different at baseline between participants who completed a 2 months follow-up survey and those who did not for the Relationships (*U* = 140.50, z = −0.646, *p* = 0.526), Communication (*U* = 161.00, z = −0.219, *p* = 0.846) or Awareness sub-scales (*U* = 135.50, *z* = −0.998, *p* = 0.330).

Participants who did not complete a 2 months follow-up survey were significantly less likely to be fairly/extremely confident immediately after the workshop to “respectfully engage with Aboriginal and Torres Strait Islander people whose attitudes and values to health are different from your own” (62%) compared with participants who did complete a 2 months follow-up survey (96%)(FET, *p* = 0.043). Scores were not significantly different at post workshop between participants who completed a 2 months follow-up survey and those who did not for the Relationships (*U* = 115.00, z = 1.058, *p* = 0.317), Communication (*U* = 118.50, z = 1.224, *p* = 0.237) or Awareness sub-scales (*U* = 102.00, *z* = 0.466, *p* = 0.674).

### Quantitative findings

#### Dimensions of cultural competency and changes over time

An Exploratory Factor Analysis used principal axis factor analysis to determine the underlying dimensions of the cultural competency items. Final estimates of communalities were iterated from squared multiple item correlations to convergence. The item pool was deemed suitable for factor analysis (KMO = 0.76). Using Kaiser’s criterion (Eigenvalues > = 1.0) together with Cattell’s scree test, four factors were extracted accounting for 76% of the common variance factor.

The instrument contained four sub-scales, with three scales labelled as Relationships, Communication and Awareness having excellent scale reliability (Relationships alpha = 0.870, Communication alpha = 0.890, Awareness alpha = 0.789). The fourth sub-scale was excluded as item six loaded on three sub-scales and had low reliability (alpha = 0.024). Factor loadings for the three sub-scales ranged from 0.58 to 0.92.

A mean score was calculated for each sub-scale from the corresponding items: Relationships calculated from items 8 and 10–13; Communication from items 1–3 and 9; and Awareness from 4, 5 and 7 (Table 2). Participants’ confidence scores to apply cultural safety in healthcare settings were significantly affected over the three time points measured in the areas of Relationships (*X* ^2^ [2, *N* = 23] = 24.602, *p* < 0.001), Communication (*X* ^2^ [2, N = 23] = 12.111, *p* = 0.002) and Awareness (*X* ^2^ [2, N = 23] = 33.778, p < 0.001) (Table 2). Pairwise comparisons showed that participants’ confidence scores significantly increased from baseline compared to immediately after the workshop for Relationships (z = −3.582, p < 0.001), Communication (z = −2.847, *p* = 0.004), and Awareness (z = −4.567, *p* < 0.001); and 2 months after the workshop for Relationships (z = −3.529, p < 0.001), Communication (z = −3.186, *p* = 0.001), and Awareness (z = −3.942, p < 0.001). There was no significant difference in cultural safety confidence scores for any sub-scale when compared between immediately after the workshop to 2 months after: Relationships (z = −1.798, *p* = 0.072), Communication (z = −0.405, *p* = 0.685) or Awareness (z = −1.396, *p* = 0.163).

**Table 2:** Cultural safety confidence score of participants for each sub-scale before, after, and two months after attending a cultural education workshop

Cultural confidence score Mean (S.D.)	Baseline (*n* = 37–38)	Post workshop (*n* = 31)	Two months post workshop (n = 25)
Relationships	1.4 (0.60)	2.0 (0.49)**	2.0 (0.39)**
Communication	1.5 (0.61)	2.0 (0.56)*	2.0 (0.44)*
Awareness	1.0 (0.58)	1.8 (0.53)**	1.8 (0.46)**

*p < 0.05 significant difference between baseline and post/two months workshop, ** *p* < 0.001 significant difference between baseline and post/two months workshop

#### Baseline

Participants’ levels of confidence in providing culturally safe healthcare to Aboriginal Australians at baseline are shown for each item in Table 3. Less than 10% of respondents were extremely confident about any of the 14 items (data not shown) and the majority of participants were not at all/a little confident in six of 14 aspects related to providing culturally safe healthcare, including not knowing the location of Aboriginal communities in rural and remote WA (87%). Most participants were fairly or extremely confident about providing culturally safe care for half of items and at least two thirds were fairly or extremely confident about interacting with Aboriginal people (71%) and initiating conversations (66%). There were no significant differences with respect to demographic variables and level of confidence (all *p* > 0.05).

**Table 3:** Participants’ confidence to apply culturally safe practices in the healthcare setting before, after and two months after attending a cultural education workshop

How confident are you…	Pre-workshop (*n* = 36–38)		Post-workshop (n = 31)		Two months follow up (*n* = 24–25)	
	Not at all/ a little bit *n*(%)	Fairly/ extremely n(%)	Not at all/ a little bit *n*(%)	Fairly/ extremely *n*(%)	Not at all/ a little bit n(%)	Fairly/ extremely *n*(%)
1… to interact with people from Aboriginal or Torres Strait Islander cultures?	11 (28.9)	27 (71.1)	6 (19.4)	25 (80.6)	3 (12.0)	22 (88.0)
2… to initiate conversations with people from Aboriginal or Torres Strait Islander cultures?	13 (34.2)	25 (65.8)	5 (16.1)	26 (83.9)	2 (8.0)	23 (92.0)
3… to talk about cancer with people from Aboriginal or Torres Strait Islander cultures?	18 (48.6)	19 (51.4)	9 (29.0)	22 (71.0)	5 (20.0)	20 (80.0)*
4… to identify your beliefs or assumptions about Aboriginal or Torres Strait Islander cultures?	30 (78.9)	8 (21.1)	10 (32.3)	21 (67.7)*	8 (32.0)	17 (68.0)*
5… to reflect on how your beliefs or assumptions influence your interactions with Aboriginal and Torres Strait Islander patients in your healthcare practice?	28 (73.7)	10 (26.3)	6 (19.4)	25 (80.6)**	6 (24.0)	19 (76.0)*
6… in your knowledge of the location of Aboriginal communities in rural and remote WA?	33 (86.8)	5 (13.2)	24 (77.4)	7 (22.6)	15 (62.5)	9 (37.5)
7… in your knowledge and understanding of the social circumstances of Aboriginal and Torres Strait Islander patients in your care?	32 (82.1)	7 (17.9)	11 (35.5)	20 (64.5)**	6 (24.0)	19 (76.0)**
8… to build trust between yourself and Aboriginal patients and their families?	22 (59.5)	15 (40.5)	6 (19.4)	25 (80.6)*	5 (20.0)	20 (80.0)*
9… to respectfully engage with Aboriginal and Torres Strait Islander people whose attitudes and values to health are different from your own?	20 (54.1)	17 (45.9)	4 (12.9)	27 (87.1)*	2 (8.0)	23 (92.0)*
10… to discern whether your communication with Aboriginal and Torres Strait Islander patients is effective or ineffective?	25 (69.4)	11 (30.6)	9 (29.0)	22 (71.0)*	11 (44.0)	14 (56.0)
11… to seek help for any problem you encounter in caring for Aboriginal and Torres Strait Islander patients?	15 (40.5)	22 (59.5)	1 (3.2)	30 (96.8)*	1 (4.0)	24 (96.0)*
12… to collaborate with Aboriginal colleagues around delivering health care to Aboriginal patients?	15 (40.5)	22 (59.5)	5 (16.1)	26 (83.9)	2 (8.0)	23 (92.0)*
13… to collaborate with non-Aboriginal colleagues around delivering health care to Aboriginal patients?	16 (44.4)	20 (55.6)	2 (6.5)	29 (93.5)*	2 (8.0)	23 (92.0)*
14… that you work in a team that delivers culturally safe care to Aboriginal and Torres Strait Islander patients?	16 (43.2)	21 (56.8)	2 (6.5)	29 (93.5)*	3 (12.0)	22 (88.0)*

*0.01 ≤ *p* < 0.05, **p < 0.01. Compared to baseline, a significantly greater proportion of participants were fairly/extremely confident about this item after the workshop

#### Post workshop

Within 2 days of attending a workshop, 82% (*n* = 32) of participants completed a post-workshop survey to rate their level of confidence in providing culturally safe care for Aboriginal people.

Over half of the participants were fairly/extremely confident about 13 of the 14 items (Table 3). Compared to baseline, a significant number of participants changed to being fairly/extremely confident about providing healthcare to Aboriginal patients for nine of 14 cultural competency items immediately after the workshop including: the identification (68%) (*X* ^2^ [1, *N* = 31] =12.071, *p* = 0.001) and influence (81%) (*X* ^2^ [1, N = 31] =15.059, *p* < 0.001) of personal beliefs and assumptions, understanding the patients’ social circumstance (64%) (*X* ^2^ [1, N = 31] =14.062, p < 0.001), building trust (81%) (*X* ^2^ [1, N = 31] =6.667, *p* = 0.010), respectful engagement (87%)(*X* ^2^ [1, N = 31] =6.667, p = 0.010), effective communication (71%) (*X* ^2^ [1, N = 31] =7.562, *p* = 0.006), seeking help for problems encountered (97%) (*X* ^2^ [1, N = 31] =7.692, p = 0.006), collaborating with colleagues (94%) (*X* ^2^ [1, N = 31] =6.667, p = 0.010) and working as a team (94%)(*X* ^2^ [1, N = 31] =6.667, p = 0.010)(Table 3).

Similar to baseline, the majority had little or no confidence in their knowledge of the locations of rural and remote Aboriginal communities (77%).

#### Two months post workshop

Two months after attending a workshop, participants were invited to complete a post-workshop survey to rate their level of confidence on providing culturally safe healthcare to Aboriginal people (Table 3). The follow-up response rate was 64% (*n* = 25).

More than three quarters of participants were fairly/extremely confident in eleven of the fourteen cultural competency items and more than half were fairly/extremely confident that they could identify their own beliefs or assumptions about Aboriginal patients (68%), and discern if their communication with Aboriginal patients is effective (56%). Sixty-two per cent (62%) were not at all/a little bit confident in knowing the location of rural and remote Aboriginal communities in WA.

Similarly to immediately after the workshop, 2 months after completing the workshop a significant number of participants changed to being fairly/extremely confident in the same 10 of 14 cultural competency items, excluding the effective communication item, compared to baseline. The items included: the identification (68%)(*X* ^2^ [1, *N* = 24] =9.600, *p* = 0.002) and influence of personal beliefs and assumptions (76%)(*X* ^2^ [1, N = 24] =12.071, *p* = 0.001), understanding patients’ social circumstances (76%)(*X* ^2^ [1, *N* = 23] =12.071, p = 0.001), building trust (80%) (*X* ^2^ [1, N = 23] =9.091, *p* = 0.003), respectful engagement (92%)(*X* ^2^ [1, N = 23] =8.100, *p* = 0.004), seeking help for problems encountered (96%) (*X* ^2^ [1, N = 23] =4.900, *p* = 0.027), collaborating with non-Aboriginal colleagues (92%)(*X* ^2^ [1, N = 23] =5.818, *p* = 0.016) and working as a team (88%)(*X* ^2^ [1, N = 23] =4.000, *p* = 0.046)(Table 3).

Unlike immediately after the workshop, by 2 months a significant number of participants changed to being fairly/extremely confident talking about cancer with people from Aboriginal cultures (80%)(*X* ^2^ [1, *N* = 23] =4.900, p = 0.027) and collaborating with Aboriginal colleagues around delivering health care to Aboriginal patients (92%)(*X* ^2^ [1, N = 23] =4.900, p = 0.027). Immediately after the workshop, a significantly greater proportion of participants were fairly/extremely confident about discerning whether their communication with Aboriginal patients was effective; however, 2 months after the workshop there was no significant difference in the proportion of fairly/extremely confident participants compared to baseline (56%)(*X* ^2^ [1, N = 23] =2.083, *p* = 0.149).

The items in which there was no significant change from baseline at any time point related to confidence about interaction (item 1) and initiating (item 2) conversation with people from Aboriginal and Torres Strait Islander cultures and about the location of communities (item 6).

#### Qualitative findings

##### Baseline

In their written responses, most participants identified good communication, respect, and awareness of cultural differences as the most important skills when relating to Aboriginal patients. A sub-theme of ‘sensitivity’ emerged from the data, relating to both the themes of communication and respect and included awareness and understanding of differences between cultures:

Appropriate verbal and non-verbal communication skills, the ability to build rapport and establish a level of mutual trust, be able to work as a team/ involve the patient’s family to provide an effective level of health care. (RT21).

Patience and understanding. Good communication - use of language appropriate to their level of understanding, if necessary, use an Aboriginal interpreter, Build trust - explain or demonstrate what you will be doing, Display a friendly demeanour - smile when you meet them for the first time. (RT12).

Cultural awareness and sensitivity were considered integral to building inter-cultural relationships with some participants identifying the social context, family and lived experience of Aboriginal people as important. Some saw Aboriginal Australians as a homogeneous rather than diverse cultural group where characteristics were essentialised or seen as immutable. Negative stereotypes still prevailed and Aboriginal people, rather than the preconceived assumptions of health providers or the health system, were seen as the problem:

They drink alcohol excessively, smoke cigarettes excessively and social skills aren’t as developed as other cultures. (RT4).

They don’t care about what is happening to their health, are disinterested in what is going on - lack trust towards professionals - don’t tend to arrive for appointments on time or at all. (RT18).

They have limited knowledge of health and nutrition. (RT14).

They are generally shy people, they are uncomfortable, unsettled or scared when taken out of their usual environment/habitat. (RT12).

Differences in cultural beliefs and expectations often led to tension with suggestions that Aboriginal people:

…are uncomfortable in the hospital environment. They are potentially untrusting of the medical staff. It is difficult to build a rapport. (RT32).

Rather than participants reflecting on their own assumptions or on the impact of the organisational structure on patients’ responses, participants’ difficulties relating to Aboriginal patients focused more on poor communication and patient ‘non-compliance’ with treatment and attendance at what are generally daily appointments for several weeks. Some participants suggested different expectations between the health professional and patient led to health professionals feeling uncertain and lacking confidence about how best to respond, particularly around the importance of continuing treatment:

One thing I’ve noticed that even after doing your best to explain things, you don’t receive any great acknowledgement from them that they understand what you have told them. You proceed with their treatment with a degree of uncertainty. This is particularly unsettling for a radiation therapist when they are meeting and treating the patient on the first day of their treatment course. You want any new patient to understand the treatment process. With Indigenous patients, I’m never really confident that this is the case. (RT12).

It has sometimes been difficult to engage [patients] in conversation, especially in order to find out how they are coping with the treatment, which has sometimes led to symptoms progressing way past the point where some intervention would be useful. It is also difficult sometimes to reinforce how important it is to come for treatment as prescribed. (RT27).

##### Post-workshop

The most important theme participants identified immediately following the workshop was the need for health professionals to treat Aboriginal patients with respect, demonstrated by clear communication, understanding their concerns and empathy towards cultural differences.

While pre-workshop responses indicated awareness of the importance of good communication and respect, post-evaluation responses demonstrated how they could be applied to practice:

Communication skills, both verbal and non-verbal; respect for elders; respect for the deceased and namesakes of deceased; respect for gender roles; understanding ‘shame’. And the ability to develop trust and rapport with patients. (RT21).

Ask the patient what country are they from. This will hopefully open the doors to good communication or at least find something that you and they may have in common to discuss e.g. children etc. Understand that they may be scared or unsettled being in a different tribal area; understand that this may be their first visit to a city; understand that they may be alone without the support of their family. (RT12).

These responses suggest an acknowledgement of the social context where the main beliefs and assumptions participants held about Aboriginal people shifted post workshop from, for example, projecting the problem of non-compliance onto Aboriginal people to participants showing a willingness to reflect on their own pre-existing beliefs and assumptions about Aboriginal people and examine their effect on practice:

That they are mostly non-compliant; that they will do what they want when they want. Since the talk I attended I am addressing these beliefs and assumptions. (RT28).

Aboriginal and Torres Strait Islander patients may be the recipients of unintentional racism. Aboriginal and Torres Strait Islander patients may feel that health practitioners are discriminating against them, when the practitioner may simply be unaware of their cultural beliefs or boundaries. (Other-43).

Other responses suggested that essentialist views about Aboriginal people still prevailed after the workshop and reflected homogeneity rather than diversity between Aboriginal people and a sense of ‘us and them’:

They are extremely spiritual people, therefore, sensitive to certain issues that do not seem matter to others. They are carefree about lives, prefer not to interfere with nature, including medical intervention of illness they suffer, they feel intimidated and uneasy at a foreign place that is not their ‘country’. (RT8).

Following the workshop, other participants reviewed their preconceptions about cultural differences and respect and how these could inform practice – this was often presented as a ‘tick box’ list:

Do not ask questions that require a yes or no answer; always have a male present when talking to a male Aboriginal or Torres Strait Islander. Patients may have the same name as someone recently deceased so we should be wary of this. Family are extremely important and could be asked about, to develop mutual respect, trust and ongoing rapport. (RT21).

The workshop led one participant to examine his/her own assumptions about the rationale for behaviour and seeking to understand the meaning of behaviour from the perspective of cultural differences:

I used to think that Aboriginal people were shy, but now I know that they often turn away and feel ‘shame’ when they don’t understand something being said to them. If you notice this, tell them that there is no shame. I used to think that all Aboriginal people could read and understand English. I have since found out that this is not the case. They may speak different dialects of Aboriginal languages ahead of English. I’ve also learnt that an Aboriginal language isn’t a written language - it is simply an oral language. That they have a large network of family support - this is sometimes not the case when they have to travel out of their normal environment to receive healthcare. (RT12).

Most participants found the workshops helpful and informative.

I think it was a great way to tackle cultural differences and cultural dangers that Aussie practitioners would probably never have thought about. They should have something like this for all types of cultural, gender, age groups. (Student RT52).

The workshops increased participants’ knowledge of Aboriginal culture and this in turn increased their confidence to interact with Aboriginal people with a view to ultimately achieve better health outcomes.

Learning the cultural differences; what is appropriate and what is not when communicating with Aboriginal people; methods on how we can try to find a link with them in order to gain their cooperation to achieve treatment goals. Such knowledge empowers me to provide a successful outcome in delivering radiation treatment. (RT8).

Participants also engaged in a process of self-reflection following the workshop:

Refreshed my knowledge with regards to how to interact and build trust with an Aboriginal patient. It also refreshed my knowledge of the different cultural differences that could impact our ability to treat the patient. For example, the case studies conducted in the workshop helped me understand what I should consider when treating older patients that could be considered elders in their communities, or the differences in culture surrounding gender. These examples have helped me feel more confident to deal with potential issues that could arise and better treat an Aboriginal patient by making them feel culturally safe. (Student RT58).

Nonetheless, responses suggested that most participants wanted a list of ‘dos and don’ts’ when they engaged with Aboriginal people:

I think the workshop may have been slightly more useful if we were told what to do in certain situation or who we could get into contact with to ensure our patients are getting the support they need if we can’t directly help them. (RT40).

##### Two months post workshop

Responses 2 months following the workshop indicated that participants had reflected on how they communicated with Aboriginal patients with most being aware of the importance of building rapport through more sensitive and respectful questioning. This included viewing Aboriginal people more holistically in the context of their culture, community, language and family and using that knowledge to help build rapport in establishing a relationship. This approach also avoided using medical jargon when explaining the treatment process and involved checking if patients had understood health providers’ instructions. As a result of the workshop some participants noticed they were less fearful of saying the wrong thing, or saying nothing. Instead they felt the knowledge they had gained increased their confidence and translated into *‘using different questions to gain trust’* (RT35) and:

Since the workshop I have treated an Aboriginal patient and felt much more at ease when addressing their concerns and assisting them to and from the treatment area. (Other-43).

Participants were more aware of ‘*finding a point of engagement’* (RT27) and being more sensitive to the needs of the Aboriginal patient:

I take a bit more time communicating with them and their family, making sure they are comfortable with the treatment, and if not, try to get someone that can talk and explain things in their language and not assume they know what you are talking about. (RT7).

As participants linked ideas they had learned from the workshop to practice, they were able to establish and build relationships with their patients and see the effect of their efforts:

A woman from Port Hedland said she was happy to come for treatment every day because we took the time to get to know her and would look after her, giving her the respect she deserved as an elder. We asked her about her family back home, children, grandchildren and great grandchildren as well as her community. (RT21).

One participant noticed that one of the challenges in linking respectful communication to practice was the small number of Aboriginal patients encountered by health professionals working in cancer care. Another noted the inappropriate literature given to patients on the side effects of chemotherapy and radiotherapy and suggested it needed to be more sensitive to cultural differences.

## Discussion

This study demonstrates that attendance at one workshop about respectfully relating to Aboriginal patients with cancer was effective not only in increasing health professionals’ confidence in applying culturally safe practices, but also appeared to translate knowledge into better care for Aboriginal patients with cancer. Improvements were evident immediately following the workshop and were sustained 2 months later.

After the workshop, an increase in reported confidence in applying culturally safe practices was found for most items (4, 5, 7–11, 13, 14) and this improvement was also found 2 months later for the same items, apart from one (10). This pattern of sustained improvement was also evident in the sub-scales identified in the instrument, Relationships, Communication and Awareness, which all improved after the workshop and persisted to 2 months after the workshop. These findings suggest that the workshop improved participants’ confidence in applying culturally safe practices for Aboriginal patients receiving cancer treatment. This supports findings from a Cochrane systematic review on cultural competence education for health professionals [23]. The review found that, despite the generally poor quality of evidence, such education can lead to improved understanding between health professionals trained in the western biomedical model and patients from culturally and linguistically diverse backgrounds.

Participants in our study highlighted the importance of communication as an important skill when treating patients that improved after the workshop and was sustained 2 months later. The workshop raised participants’ awareness about what helped or hindered good communication and the importance of reflecting on their current practice. Communication was also identified as a sub-scale of the quantitative items about implementing culturally safe practices and this was similarly improved after the workshop and sustained 2 months later.

Knowledge and confidence to apply respectful communication increased in participants and some demonstrated how it was subsequently applied to practice. Interestingly, two items which involve talking about cancer with Aboriginal patients (3) and collaborating with Aboriginal colleagues (12) significantly improved only after 2 months. These are less common activities in the usual daily tasks of RTs, and it is likely 2 months were needed to experience these activities before improved confidence could be determined by participants. This finding suggests that with time in clinical practice, some participants were able to apply their knowledge and practise communication skills, and determine whether they were effective. One item in the relationships sub-scale, confidence to discern whether communication with Aboriginal patients is effective or ineffective, significantly improved immediately after the workshop; however, improvement was not sustained 2 months later. This may reflect the beginning of a decline in confidence as time after the workshop passed and which may be due to reduced or no opportunities to apply knowledge to practice with Aboriginal patients.

Participants’ willingness to engage with the concept of reflective practice was noted in responses which recognised the tendency to externalise the problem for example, of non-compliance with treatment, onto Aboriginal patients, rather than examining any negative preconceptions held about Aboriginal people that might inform their interactions and compromise care. In the evaluation 2 months following the workshop, awareness of the consequences of negatively judging Aboriginal patients had led some participants to change their practice to be more sensitive and respectful of Aboriginal patients in their care. This reflective theme was similar to the quantitative measure of Awareness which improved immediately after the workshop and sustained 2 months later.

Nonetheless we note that, while some participants became aware of the limitations of their own knowledge of Aboriginal culture, and wanted to know more to improve their relationships with Aboriginal patients, others still wanted a ‘tick box’ of what to do in specific contexts. Most participants acknowledged the importance of the social and cultural context when caring for Aboriginal patients to better understand their lived experience although were unaware of remote locations where some of their patients lived. Little confidence to interact (item 1) and initiate conversations (item 2) with Aboriginal people were the only items which did not improve. However, this lack of knowledge about where the patient’s community was located (item 6) also provided opportunities for health professionals to ‘break the ice’ with their Aboriginal patients and learn about where they lived. This could help build their confidence about starting a conversation as well as increasing their knowledge of geographical locations of communities. In addition, education to improve communication and learn where Aboriginal communities in WA are located, particularly for those who have moved from interstate or overseas, could reinforce this process [24, 25]. After the workshop, some participants continued to view Aboriginal culture across Australia as homogeneous rather than diverse and the tendency persisted to essentialise Aboriginal people’s characteristics and negatively stereotype them. Findings highlighted a need for Aboriginal and non-Aboriginal people to work together to improve differentials in cancer outcomes for Aboriginal people [26].

This project focused on health professionals reflecting on delivering care respectful of Aboriginal patients with cancer. The workshop also acknowledged broader socio-cultural and structural barriers influencing Aboriginal patients making optimum choices about their care. These included factors impacting on access to services such as discrimination and competing demands on limited budgets prioritised over cancer care. It is important that health professionals acknowledge and respond sensitively to such barriers so they can provide culturally safe care in mainstream health services [26, 27].

## Limitations

This evaluation was limited to the Perth metropolitan area where discrete workshops were offered at two locations; however, all radiation oncology staff at all metropolitan sites of the public and private service providers were invited to attend the workshop. While small numbers limited statistical analysis, the participation rate of workshop attendees in the study was good (66%) compared to other surveys of RTs [28]. It should also be noted that participation decreased between survey rates with attrition rates of 18% post-workshop and 36% 2 months later. A sensitivity analysis found a bias due to drop-outs with three instances in which participants who dropped out were significantly less likely to be fairly/extremely confident for that item at an earlier time point. The three items belong to the communication sub-scale, which itself was also significantly different at baseline between participants who continued and those who dropped out, and may suggest that participants who dropped out did not have opportunities or willingness to interact with patients from an Aboriginal and Torres Strait Islander culture and their discontinued participation may have related to this. Despite limitations, this initial study demonstrates that this workshop was useful to participants and highlights that it might be beneficial to offer further workshops like this to radiation oncology professionals and other health professionals working with Aboriginal cancer patients. It would also be beneficial to conduct research to determine patient and carer perceptions before and after health professional education is provided. It should also be noted that the evaluation did not measure changes to practice beyond 2 months so it is unknown whether improvements were sustained.

## Conclusion

Our findings suggest that one workshop on providing culturally safe care for Aboriginal patients with cancer can increase health professionals’ confidence, knowledge of Aboriginal patients’ culture and lived experience and lead to more respectful relationships and sensitive practice. While this supports other findings that education or training in delivering health care respectful of cultural differences can improve relationships between health professionals trained in the western biomedical model of health care and patients from culturally and linguistically diverse backgrounds [23], we interpret our findings cautiously given small numbers and participants’ self-reporting. Nonetheless, we support other recommendations that such programs, including ‘one-off’ programs encouraging reflective practice and challenging negative stereotypes about Aboriginal Australians are integrated into organisational practice within a broader workplace culture as a strategy to implement culturally safe care [23, 29].

Improved experiences of cancer care for Aboriginal patients is likely to translate to more timely access to services and improved cancer survival rates. Implementing cultural education for health professionals that translates into better practice as part of ongoing professional development is a contributing factor to achieving this goal. Partnerships between Aboriginal and non-Aboriginal stakeholders in which feedback from the Aboriginal community about their responses to care, what works and what needs to change, are also integral to improving health care and can be instrumental in changing practice and increasing access to services [30, 31]. We suggest taking such factors into account is necessary if widespread improvements in the care offered to Aboriginal patients with cancer is to occur.

## Additional files

Additional file 1: Working together to improve healthcare for Aboriginal and Torres Strait Islander Australians Pre- workshop questionnaire. Description: Pre-workshop questionnaire. (DOCX 28 kb)

Additional file 2: Working together to improve healthcare for Aboriginal and Torres Strait Islander Australians Post-workshop questionnaire. Description: Post-workshop questionnaire. (DOCX 22 kb)

Additional file 3: Working together to improve healthcare for Aboriginal and Torres Strait Islander Australians two mons post-workshop questionnaire. Description: Two months post-workshop questionnaire. (DOCX 17 kb)

## Abbreviations

FET: Fisher’s exact tests
IBM SPSS: International business machines corporation statistical package for the social sciences
KMO: Kaiser-meyer-olkin measure of sampling adequacy
NK: Not known
RON: Radiation oncology nurse
RT: Radiation therapist
WA: Western Australia
