# Supplementary material for: Does one workshop on respecting cultural differences increase health professionals’ confidence to improve the care of Australian Aboriginal patients with cancer? An evaluation
Source: BMC Health Serv Res. 2017 Sep 15;17:660. doi: 10.1186/s12913-017-2599-z (PMC5603013; doi:10.1186/s12913-017-2599-z)
Supplement: Supplementary file 1 — Working together to improve healthcare for Aboriginal and Torres Strait Islander Australians Pre- workshop questionnaire. Description: Pre-workshop questionnaire. (DOCX 28 kb) [file 12913_2017_2599_MOESM1_ESM.docx]

**Working together to improve healthcare for Aboriginal and Torres Strait Islander Australians**

**Pre- workshop questionnaire**

Dear Workshop Participant

Thank you for taking the time to complete this questionnaire. It should take about 15 minutes.

These questions are designed to measure changes in confidence after completing the workshop on culturally safe health care to Aboriginal and Torres Strait Islander patients. We will ask you to complete the questionnaire again at the end of the workshop to see if the workshop content increased your confidence to care for Aboriginal people in culturally safe ways. Your confidentiality will be protected and your name or any identifying details will not be used in reports or publications

Thanks again.

Professor Marion Kickett and Associate Professor Angela Durey

________________

**Demographics**

**Do you identify as Aboriginal or Torres Strait Islander?** □ Yes □ No

**Age group** □20-30 □31-40 □41-50 □51-60 □ 60+

**Gender** Male □ Female □

W**hat is your profession?** □ radiation therapist □ radiation oncology nurse □ medical practitioner

□ social worker □ radiographer □ Student Master of RT

□ physiotherapist □ Aboriginal Health Worker

□ other (please name)____________________________

**Please list your qualifications:** □ Certificate___________________________________

□ Diploma_____________________________________

□ Bachelor degree_______________________________

□ Masters degree_______________________________

□ Other_______________________________________

□ Other_______________________________________

**What year did you start working independently in your health profession? ____________**

**Where did you train as a health professional?**

□ Western Australia □ New South Wales □ Queensland □ South Australia

□ Victoria □ Tasmania □ Australian Capital Territory □ North Territory

□Overseas (please name country)______________

**Do you have Aboriginal or Torres Strait Islander patients in your case load?** □ Yes □ No □Don’t know

**Have you undergone Aboriginal cultural education and training before?**

□ Yes. Please describe what this involved_________________________ □ No

**PART 1**

| **How confident are you….** | **Not at all confident** | **A little bit confident** | **Fairly confident** | **Extremely confident** |
| --- | --- | --- | --- | --- |
| 1. …to interact with people from Aboriginal or Torres Strait Islander cultures? |  |  |  |  |
| 1. ...to initiate conversations with people from Aboriginal or Torres Strait Islander cultures? |  |  |  |  |
| 1. …to talk about cancer with people from Aboriginal or Torres Strait Islander cultures? |  |  |  |  |
| 1. ...to identify your beliefs or assumptions about Aboriginal and Torres Strait Islander people? |  |  |  |  |
| 1. ...to reflect on how your beliefs or assumptions influence your interactions with Aboriginal and Torres Strait Islander patients in your healthcare practice? |  |  |  |  |
| 1. … in your knowledge of the location of Aboriginal communities in rural and remote WA? |  |  |  |  |
| 1. ...in your knowledge and understanding of the social circumstances of Aboriginal and Torres Strait Islander patients in your care? |  |  |  |  |
| 1. ...to build trust between yourself and Aboriginal patients and their families? |  |  |  |  |
| 1. ...to respectfully engage with Aboriginal and Torres Strait Islander people whose attitudes and values to health are different from your own? |  |  |  |  |
| 1. ...to discern whether your communication with Aboriginal and Torres Strait Islander patients is effective or ineffective? |  |  |  |  |
| 1. ...to seek help for any problems you encounter in caring for Aboriginal and Torres Strait Islander patients? |  |  |  |  |
| 1. ...to collaborate with Aboriginal colleagues around delivering health care to Aboriginal patients? |  |  |  |  |
| 1. ...to collaborate with non-Aboriginal colleagues around delivering health care to Aboriginal patients? |  |  |  |  |
| 1. ...that you work in a team that delivers culturally safe care to Aboriginal and Torres Strait Islander patients? |  |  |  |  |

**Please list 3 to 5 important skills that you think should be demonstrated by health practitioners when treating patients of an Aboriginal or Torres Strait Islander background:**

|  |
| --- |
|  |
|  |
|  |
|  |
|  |
|  |
|  |

**Please list 3 to 5 beliefs or assumptions that you have about Aboriginal or Torres Strait Islander cultures:**

|  |
| --- |
|  |
|  |
|  |
|  |
|  |
|  |
|  |

**Have you previously had difficulty interacting with patients of Aboriginal or Torres Strait Islander cultures? Please describe any difficulties**

|  |
| --- |
|  |
|  |
|  |
|  |
|  |
|  |

*Thank you for your participation*
