# Supplementary material for: Does one workshop on respecting cultural differences increase health professionals’ confidence to improve the care of Australian Aboriginal patients with cancer? An evaluation
Source: BMC Health Serv Res. 2017 Sep 15;17:660. doi: 10.1186/s12913-017-2599-z (PMC5603013; doi:10.1186/s12913-017-2599-z)
Supplement: Supplementary file 2 — Working together to improve healthcare for Aboriginal and Torres Strait Islander Australians Post-workshop questionnaire. Description: Post-workshop questionnaire. (DOCX 22 kb) [file 12913_2017_2599_MOESM2_ESM.docx]

**Working together to improve healthcare for Aboriginal and Torres Strait Islander Australians**

**post-workshop questionnaire**

| **How confident are you….** | **Not at all confident** | **A little bit confident** | **Fairly confident** | **Extremely confident** |
| --- | --- | --- | --- | --- |
| 1. …to interact with people from Aboriginal or Torres Strait Islander cultures? |  |  |  |  |
| 1. ...to initiate conversations with people from Aboriginal or Torres Strait Islander cultures? |  |  |  |  |
| 1. …to talk about cancer with people from Aboriginal or Torres Strait Islander cultures? |  |  |  |  |
| 1. ...to identify your beliefs or assumptions about Aboriginal and Torres Strait Islander people? |  |  |  |  |
| 1. ...to reflect on how your beliefs or assumptions influence your interactions with Aboriginal and Torres Strait Islander patients in your healthcare practice? |  |  |  |  |
| 1. … in your knowledge of the location of Aboriginal communities in rural and remote WA? |  |  |  |  |
| 1. ...in your knowledge and understanding of the social circumstances of Aboriginal and Torres Strait Islander patients in your care? |  |  |  |  |
| 1. ...to build trust between yourself and Aboriginal patients and their families? |  |  |  |  |
| 1. ...to respectfully engage with Aboriginal and Torres Strait Islander people whose attitudes and values to health are different from your own? |  |  |  |  |
| 1. ...to discern whether your communication with Aboriginal and Torres Strait Islander patients is effective or ineffective? |  |  |  |  |
| 1. ...to seek help for any problems you encounter in caring for Aboriginal and Torres Strait Islander patients? |  |  |  |  |
| 1. ...to collaborate with Aboriginal colleagues around delivering health care to Aboriginal patients? |  |  |  |  |
| 1. ...to collaborate with non-Aboriginal colleagues around delivering health care to Aboriginal patients? |  |  |  |  |
| 1. ...that you work in a team that delivers culturally safe care to Aboriginal and Torres Strait Islander patients? |  |  |  |  |

*Please show your level of agreement with the following statements by ticking* (🗹) *the appropriate box.*

|  | **Strongly disagree** | **Disagree** | **Unsure** | **Agree** | **Strongly Agree** |
| --- | --- | --- | --- | --- | --- |
| The workshop was informative |  |  |  |  |  |
| The information was well presented |  |  |  |  |  |
| The facilities- venue and catering were appropriate |  |  |  |  |  |
| The organisation of the activity was efficient |  |  |  |  |  |
| The duration of the activity was appropriate |  |  |  |  |  |
| I would like to take part in similar CPD activities |  |  |  |  |  |

| \| **Please rate the degree to which your own learning needs were met:** \| \| \| \| --- \| --- \| --- \| \| □ Entirely met \| □ Partially met \| □ Not met \| \| **Please rate the degree to which this activity was relevant to your own practice:** \| \| \| \| □ Entirely relevant \| □ Partially relevant \| □ Not relevant \|   What is the most significant learning you take away from participating in this activity and why?  _____________________________________________________________________________________________  _____________________________________________________________________________________________  _____________________________________________________________________________________________  Do you have any comments about this activity?  _____________________________________________________________________________________________  _____________________________________________________________________________________________  _____________________________________________________________________________________________  Are there any ways in which you think this activity could be improved?  _____________________________________________________________________________________________  _____________________________________________________________________________________________  _____________________________________________________________________________________________ |
| --- | --- | --- | --- | --- | --- | --- | --- | --- | --- | --- | --- | --- |
