# Supplementary material for: Does one workshop on respecting cultural differences increase health professionals’ confidence to improve the care of Australian Aboriginal patients with cancer? An evaluation
Source: BMC Health Serv Res. 2017 Sep 15;17:660. doi: 10.1186/s12913-017-2599-z (PMC5603013; doi:10.1186/s12913-017-2599-z)
Supplement: Supplementary file 3 — Working together to improve healthcare for Aboriginal and Torres Strait Islander Australians two mons post-workshop questionnaire. Description: Two months post-workshop questionnaire. (DOCX 17 kb) [file 12913_2017_2599_MOESM3_ESM.docx]

**Working together to improve healthcare for Aboriginal and Torres Strait Islander Australians**

**two months post-workshop questionnaire**

**PART 1**

| **How confident are you….** | **Not at all confident** | **A little bit confident** | **Fairly confident** | **Extremely confident** |
| --- | --- | --- | --- | --- |
| 1. …to interact with people from Aboriginal or Torres Strait Islander cultures? |  |  |  |  |
| 1. ...to initiate conversations with people from Aboriginal or Torres Strait Islander cultures? |  |  |  |  |
| 1. …to talk about cancer with people from Aboriginal or Torres Strait Islander cultures? |  |  |  |  |
| 1. ...to identify your beliefs or assumptions about Aboriginal and Torres Strait Islander people? |  |  |  |  |
| 1. ...to reflect on how your beliefs or assumptions influence your interactions with Aboriginal and Torres Strait Islander patients in your healthcare practice? |  |  |  |  |
| 1. … in your knowledge of the location of Aboriginal communities in rural and remote WA? |  |  |  |  |
| 1. ...in your knowledge and understanding of the social circumstances of Aboriginal and Torres Strait Islander patients in your care? |  |  |  |  |
| 1. ...to build trust between yourself and Aboriginal patients and their families? |  |  |  |  |
| 1. ...to respectfully engage with Aboriginal and Torres Strait Islander people whose attitudes and values to health are different from your own? |  |  |  |  |
| 1. ...to discern whether your communication with Aboriginal and Torres Strait Islander patients is effective or ineffective? |  |  |  |  |
| 1. ...to seek help for any problems you encounter in caring for Aboriginal and Torres Strait Islander patients? |  |  |  |  |
| 1. ...to collaborate with Aboriginal colleagues around delivering health care to Aboriginal patients? |  |  |  |  |
| 1. ...to collaborate with non-Aboriginal colleagues around delivering health care to Aboriginal patients? |  |  |  |  |
| 1. ...that you work in a team that delivers culturally safe care to Aboriginal and Torres Strait Islander patients? |  |  |  |  |

What did you learn from the workshop that was significant for you?

______________________________________________________________________________

______________________________________________________________________________

______________________________________________________________________________

How has that learning influenced how you now care for Aboriginal patients? (Give an example)

______________________________________________________________________________

______________________________________________________________________________

______________________________________________________________________________

What has helped you link that learning to practice?

______________________________________________________________________________

______________________________________________________________________________

______________________________________________________________________________

Have there been any challenges linking what you have learnt to practice?

______________________________________________________________________________

______________________________________________________________________________

______________________________________________________________________________

Any further comments?

______________________________________________________________________________

______________________________________________________________________________

______________________________________________________________________________
